# Supplementary material for: Content-rich biological network constructed by mining PubMed abstracts
Source: BMC Bioinformatics. 2004 Oct 8;5:147. doi: 10.1186/1471-2105-5-147 (PMC528731; doi:10.1186/1471-2105-5-147)
Supplement: Additional File 5 — The original Chilibot query results of the term "long-term potentiation (LTP)" and 22 other terms, limiting the latest references analyzed to the years 1990, 1995, 2000, and 2004. [file 1471-2105-5-147-S5.bz2 › chilibotAdditionalFile5/ltp1995/html/AMPA_ACTIN.html]

 


 **AMPA** and **ACTIN** 
  
Found 2 abstracts in PubMed,  **2 abstracts were retrieved and analyzed**.  


---

 Search Google  |
 PDF files only 
|  EDU domain only 

---

- Brain Res, 1994   The cerebral cortex is the primary source of glutamatergic afferents to the neostriatum.
  We used in situ hybridization to examine the effect of removal of the glutamatergic input to the striatum by unilateral frontal cortical ablation on the expression of genes encoding subunits from three families of glutamate receptors N methyl D aspartate receptors .
  alpha amino 3 hydroxy 5 methyl 4 isoxazolepropionate receptors .
  and metabotropic receptors .
  Significant changes were restricted to the dorsolateral quadrant of the ipsilateral striatum, the main projection area of the sensorimotor cortex.
  The expression of those messages which are normally abundant, NMDAR1, NMDAR2A, GluR1 4 flop and mGluR and 5, was decreased in the deafferented dorsolateral striatum by 10 39% at 3 days after cortical ablation and subsequently increased to 120 165% of control at 15 and 60 days.
  mRNAs encoding the flip isoforms of GluR1 4, mGluR2 and 4, and an alternatively spliced region of NMDAR1 which are undetectable or present at low levels in the striatum were not induced by cortical ablation.
  In contrast, both glial fibrillary acid protein and beta **actin** mRNA expression were markedly enhanced at 3 and 15 days, returning to near normal at 60 days.
  Striatal NMDA, **AMPA** and metabotropic type 1 ligand binding sites were increased as early as 3 days after cortical ablation, reached a peak at 15 days and remained increased for up to 60 days, while metabotropic type 2 binding was slightly but significantly reduced at 3 and 15 days and 3H kainate binding did not change significantly.
  These results demonstrate that cortical ablation, and subsequent loss of glutamatergic afferents to the striatum, results in alterations in the expression of genes encoding glutamate receptor subunits in striatal neurons.
  The regulation of these genes appears to be coordinate, so that the relative abundance of the different messages is preserved.

  - Neuron, 1993   **Calcium induced **actin** depolymerization reduces NMDA channel activity.** .
    **Actin** filaments are highly concentrated in postsynaptic densities at central excitatory synapses, but their influence on postsynaptic glutamate receptors is unknown.
    We tested whether **actin** depolymerization influences NMDA channel activity in whole cell recording on cultured hippocampal neurons.
    The ATP and calcium dependent rundown of NMDA channels was prevented when **actin** depolymerization was blocked by phalloidin.
    Rundown of **AMPA** kainate receptors was unaffected by phalloidin.
    Cytochalasins, which enhance **actin** ATP hydrolysis, induced NMDA channel rundown.
    . . . taxol or colchicine, which stabilize or disrupt microtubule assembly, had no effect.
    Protease inhibitors also had no effect.
    Our results suggest that calcium and ATP can influence NMDA channel activity by altering the state of **actin** polymerization and are consistent with a proposed model in which **actin** filaments compartmentalize a channel regulatory protein.
